# Changing health systems—advancing justice and sustainability through research, policy and practice

**DOI:** 10.1093/heapol/czag038

**Published:** 2026-06-29

**Authors:** Stephanie M Topp, Prashanth N. Srinivas, Neha Batura, Eleanor Whyle, Kabir Sheikh

**Affiliations:** Centre for Rural Remote and Tropical Health Systems, College of Medicine and Dentistry, 1 James Cook Drive, James Cook University, Townsville, 4811, Queensland, Australia; Center for Health Systems, Institute of Public Health, Bengaluru, Karnataka, 560070, India; Global Business School for Health, University College London, 7 Sidings St, London, E20 2AE, United Kingdom; School of Public Health and Family Medicine, University of Cape Town, Cape Town, 7701, South Africa; Global Business School for Health, University College London, 7 Sidings St, London, E20 2AE, United Kingdom

Health systems across the world are being reshaped by intersecting pressures—geopolitical instability, economic disruption, climate and environmental change, demographic transitions, and evolving governance arrangements. These forces are testing health system performance but also raising fundamental questions about how justice and sustainability should be factored into the way health systems are organized, financed, governed, and experienced.

Change in health systems is brokered across multiple arenas, from ministries and local governments, professional bodies and community organizations and frontline services, philanthropies, and non-profit organizations to private industry including health-related corporations and private equity firms, where actors interpret emerging pressures and respond through adaptation, innovation, and reform. Health systems therefore evolve through a continuous process of negotiation and institutional reshaping, in which efforts to address new challenges may either advance or constrain commitments to justice and sustainability. Understanding these dynamics requires attention to both formal policy frameworks and the way different actors engage with, interpret, and implement change in practice. While scholarship has long-documented system injustices, sustainability failures, and systemic inequities, there remains a need to better document and understand how health systems are adapting to disruption, how change is negotiated and implemented in practice, and how research can contribute to more just and sustainable futures.

The papers in this supplement—part of the legacy of the 8th Global Symposium on Health Systems Research in Nagasaki 2024 (Health Systems Global 2024) engage with these questions by examining how health systems respond to and shape change across diverse contexts, including informal urban settlements, decentralized governance reforms, sanction-constrained economies, and environmentally vulnerable settings. Drawing on a range of analytical and methodological approaches within health policy and systems research (HPSR), the papers explore how governance arrangements, institutional incentives, and knowledge practices influence reform trajectories in practice. The discussion that follows draws out several cross-cutting themes from the collection that characterize justice and sustainability not as static policy goals but as processes negotiated through institutional arrangements, political economy dynamics, everyday implementation realities, as well as our own reflexive research practice.

## Responding to change: how health systems negotiate uncertainty and disruption

Health systems are constantly required to respond to shocks, shifting political priorities, and evolving population needs. Several papers in this supplement highlight how system actors navigate these pressures through adaptive governance arrangements and institutional redesign.

Aktar et al. (2026) examine access to water and related health services in informal settlements in Dhaka, where service provision depends less on formal policy entitlements than on negotiated relationships among community leaders, NGOs, political brokers, and municipal actors. Residents often secure services through patron–client ties and informal payments, while NGOs mediate between communities and state institutions. These hybrid governance arrangements illustrate how health systems frequently respond to institutional gaps through locally negotiated solutions. At the same time, the findings show that such arrangements can reproduce inequities through selective participation and politicized access.

Tikkanen et al. (2026) highlight how systems respond to structural political change through institutional redesign. Drawing on interviews and focus groups across four districts, the authors find that Nepal’s federal transition has shifted planning authority and some budget discretion to municipalities, enabling locally tailored supervision and incentive structures for Female Community Health Volunteers (FCHVs). Yet, this expanded decision-space remains constrained by conditional federal grants, limited provincial capacity, and blurred accountability across tiers. FCHVs navigate multiple reporting lines—municipal, provincial, and federal—while unions struggle for formal recognition. The study provides fresh evidence of the way decentralization redistributes decision-making responsibilities, without necessarily redistributing power, complicating efforts to embed justice and sustainability in workforce governance.

Extending the focus from institutional redesign to system response under geopolitical and economic shocks, Majdzadeh and Sajadi (2026) analyse how Iran’s Health Transformation Plan faltered when economic sanctions disrupted currency flows, pharmaceutical imports, and insurance financing. Using policy-cycle analysis combined with an expanded theory-of-change framework, they identify specific breakpoints: untested assumptions about stable oil revenues, fragmented insurance pooling, weak coordination between ministries, and the absence of formal risk appraisal mechanisms. While emergency coping strategies mitigated some impacts, the absence of institutionalized learning and risk appraisal mechanisms limited the reform’s resilience.

A complementary commentary from Jalaghonia et al. (2026) extends the discussion by examining how innovation is being mobilized in disaster-prone health systems. Drawing on innovation-focused sessions from the 8th Global Symposium on Health Systems Research (HSR2024), the authors explore how digital platforms, AI-enabled decision tools, and community-driven approaches are being used to strengthen resilience and support more responsive decision-making under conditions of repeated shock. Examples ranging from community-designed systems in informal settlements to integrated health data platforms and climate-linked early warning systems illustrate the potential of these innovations to improve visibility of marginalized populations, strengthen coordination, and support anticipatory action. At the same time, the commentary cautions that technological innovation alone cannot deliver more just and sustainable systems; its contribution depends on governance arrangements that ensure accountability, inclusive participation, and equitable distribution of decision-making authority.

These papers each point to the way health systems must not only respond to shocks but also embed anticipatory governance and adaptive learning within reform design. Adaptive governance, negotiation across institutional boundaries, and pragmatic improvisation emerge as central features of system capability.

## Shaping change: innovation, reform, and the politics of institutional design

Health system change is not limited to adaptation in the face of external pressures. It also involves deliberate attempts by different actors to redesign institutions, policies, and service arrangements, and several contributions examine how these reform processes unfold within complex and context-specific political and institutional environments. Importantly, agency in these processes does not rest with public sector actors alone; professional groups, frontline workers, community organizations, and households all shape how reforms are interpreted, negotiated, and implemented in practice. Moreover, while the cases discussed here focus primarily on public sector and community-led initiatives, health system change is influenced by a far broader constellation of actors—including private providers, civil society organizations, and professional associations—whose roles are less often foregrounded. Recognizing this plurality of actors and change brokers is essential for understanding how reforms unfold and for identifying pathways through which justice and sustainability can be advanced within complex and evolving health systems.

Nair et al. (2026) analyse proposed health workforce reforms in Meghalaya, India, focusing on the challenge of addressing both specialist shortages and rising non-communicable disease burdens. Through prospective policy analysis, the study explores alternative workforce deployment models, training pipelines, and incentive structures through an explicitly political and institutional lens. The analysis highlights how reform trajectories are shaped not only, or even primarily, by epidemiological need but by fiscal constraints, professional hierarchies, and interdepartmental coordination dynamics. Decisions about recruitment rules, deployment incentives, and financing commitments ultimately determine whether reforms expand equitable access to services or reproduce existing distributional imbalances.

Jith et al. (2026) shift attention to the micro-level implementation of nutrition interventions in riverine Assam where environmental instability, including flooding and erosion continually reshapes service delivery. Frontline workers rely on informal coordination mechanisms and support from extended family networks to sustain programme delivery. The authors use the concept of ‘frontline families’ to highlight how household labour and informal social infrastructures effectively subsidize public service provision, highlighting how such arrangements enable programmes to function under difficult conditions and also raise important questions about equity and sustainability when system performance relies on unpaid or precarious labour. Village committees, for example, although formally participatory, are often shaped by caste, gender, and political hierarchies, influencing whose needs are prioritized. The findings reposition sustainability as contingent on relational labour, ecological adaptation, and negotiated governance at the micro-level.

## Decentring global narratives of change

Across the collection, a consistent theme is the central role of national and subnational arenas in shaping consequential health system change. While global institutions, financing mechanisms, and normative agendas remain influential, the papers demonstrate that the practical dynamics of reform—how policies and services are interpreted, negotiated, and enacted—are largely shaped within domestic political, institutional, cultural, and geographic contexts. This perspective reflects a long-standing orientation within HPSR, which views health systems as socially and politically embedded institutions shaped by national governance arrangements, administrative traditions, and local political economies. By directing analytical attention to these arenas, the field has challenged technocratic models of reform that assume policies or programmes designed externally or at global level can be transferred seamlessly across contexts.

In doing so, this special collection offers a corrective to the tendency in contemporary global health discourse to privilege global architectures—multilateral initiatives, international financing mechanisms, and transnational policy agendas—as the primary drivers of system change. The papers instead foreground the role of domestic institutions and dynamics, showing how national and local processes shape justice and sustainability outcomes by influencing whether and how reforms expand access, redistribute resources, or reinforce existing inequities. Taken together, the collection reaffirms the value of empirically grounded, contextually attentive research and a core insight of HPSR: that advancing justice and sustainability depends on how policies are negotiated, institutionalized, and enacted within the settings where health systems operate.

## Reframing knowledge and method: how HPSR contributes to system transformation

Finally, this special collection reflects on the role of research itself in shaping health system change, with two methodological contributions. Aivalli and colleagues (2026) present a practical guide to conducting realist evaluation in ways that foreground epistemic justice. Drawing on empirical examples from India and Zambia, the authors show how each stage of the realist cycle—from programme theory development to data interpretation and dissemination—can either reinforce or challenge hierarchies of knowledge. Their analysis emphasizes that methodological rigour in complex systems research requires deliberate attention to whose knowledge is recognized and how different actors participate in theory building and explanation. Importantly, the authors caution that realist evaluation does not inherently produce justice-oriented knowledge; rather, it functions as a versatile methodological toolkit whose emancipatory potential depends on reflexive choices about stakeholder engagement, theory elicitation, informal validation strategies, and locally grounded dissemination. In moving beyond abstract endorsement of participation, and towards concrete methodological guidance, the paper positions realist evaluation as a means of redistributing epistemic authority; shifting programme theory development from elite policy actors towards frontline implementers and community actors. It further reframes methodological rigour not as ‘neutrality’ but as deliberate engagement with power, positionality, and the politics of knowledge production.

Broadening the methodological gaze from epistemic authority within research processes to the distribution of political and institutional power within health systems, Karuveettil et al. (2026) turn attention to the analytical frameworks used to explain why health system reforms unfold as they do, conducting a scoping review of political economy analysis (PEA) in health systems research. Drawing on 28 studies, the authors show that while PEA is frequently invoked to explain reform feasibility and implementation barriers, its conceptual foundations and analytical depth vary considerably. By synthesizing definitions, analytical lenses, and reported outcomes, the paper provides practical guidance for more rigorous and explicit use of PEA in health systems research. Within this supplement, it sharpens the analytical lens on power, institutions, and political feasibility as central to advancing justice and sustainability in health system transformation.

## Future directions: justice and sustainability in changing health systems

Across diverse contexts considered by papers in this collection a common lesson emerges: justice and sustainability are negotiated through power relations and institutional practice. Reform does not unfold linearly from policy design to outcome. It is mediated by national and subnational actors who interpret, adapt, resist, and reshape policy within historically embedded structures. Taken together, therefore, the papers in this collection suggest that advancing justice and sustainability will depend on understanding how emerging forces are reshaping the political and institutional conditions under which health system reform unfolds. In this sense, the collection should also prompt attention to future inquiry. Recognizing much ongoing work, questions remain about how climate-related disruptions will reshape health system governance and financing, how digital transformations may alter the distribution of power within health systems, and how new forms of public-private engagement will affect equity and sustainability. The collection also highlights a broader pattern within HPSR: despite the growing influence of private providers, financiers and technology firms in shaping health systems, their role in driving or constraining reform trajectories remains comparatively underexamined. Given the growing influence of private providers, financiers and technology firms in many health systems, further scholarship examining how these actors shape reform trajectories—and with what implications for justice and sustainability—is essential. Further research is also needed to examine how justice-oriented reforms can be sustained over time, particularly in contexts characterized by fiscal volatility, political instability, or environmental stress.

By examining how systems respond to disruption, how actors attempt to shape reform, and how research itself can engage more critically with these processes, this collection contributes to a deeper understanding of how health systems evolve under conditions of uncertainty and contestation—and where future efforts to advance justice and sustainability must focus.

## Data Availability

No new data were generated or analysed in support of this work.
